# Fracture and Posterior Dislocation of Meniscal Bearing Insert in Mobile Bearing Unicompartmental Knee Arthroplasty: A Case Report

**DOI:** 10.5704/MOJ.1807.013

**Published:** 2018-07

**Authors:** A Munjal

**Affiliations:** Department of Orthopaedics, Aneurin Bevan University Health Board, Newport, United Kingdom

**Keywords:** unicompartmental knee arthroplasty, meniscal bearing, meniscal insert fracture, dislocation

## Abstract

Dislocation of meniscal bearing insert is a rare but well-recognised complication in meniscal bearing unicompartmental knee arthroplasty (UKA). On the other hand, fracture of meniscal bearing insert of phase III Oxford UKA has only been reported once in the current literature. The authors report a case of fracture and posterior dislocation of one of the fragments of the meniscal bearing insert in a mobile bearing medial UKA. The fracture was only diagnosed during the revision surgery. The posteriorly dislodged fragment was subsequently retrieved through the same skin incision and a new polyethylene insert of the same size was implanted.

## Introduction

Meniscal bearing Oxford UKA has been used effectively for isolated medial compartmental osteoarthritis since 19821. It achieves near-normal knee kinematics by using an unconstrained fully congruent mobile bearing polyethylene insert, which functionally mimics a natural meniscus^1,2^. This polyethylene insert is fully congruent throughout the range of motion because of a spherical femoral component^2^. This principle of congruent mobile bearing has delivered reliable long-term results for Oxford medial UKA with very low polyethylene wear rate and survival rates of 95% at 10 years^1^. But this also brings with it potential complications like dislocation or fracture of meniscal bearing insert. Dislocation rate of meniscal bearing has been reported as 0.5% for Oxford medial UKA^1^ on the contrary, fracture of meniscal insert in a phase III Oxford UKA has only been reported once in the literature^3^. We report a case of fracture of meniscal bearing in Oxford medial UKA with the dislocation of one of the fragments into the posterior capsule requiring revision surgery to retrieve and to replace the broken meniscal bearing insert.

## Case Report

A 60-year old female underwent a phase III Oxford UKA [Biomet UK Ltd, Bridgend, United Kingdom] in 2006 for antero-medial osteoarthritis. A minimally invasive medial para-patellar approach was used and medium sized femur, 44 X 28mm tibia and 3mm meniscal bearing insert were implanted. She had an uneventful post-operative recovery. She was completely asymptomatic and was discharged from the follow-up at two years following the surgery with no symptoms and a range of motion of 0 to 130 degrees.

**Fig. 1:**
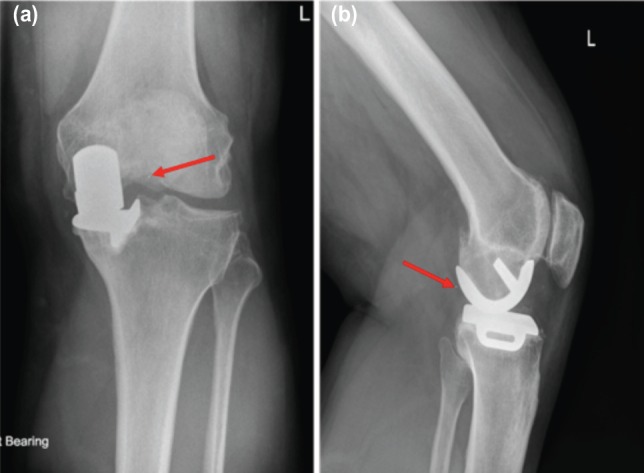
(a) AP and (b) Lateral radiographs with arrows showing dislocated meniscal bearing insert.

She presented to the Accident and Emergency (A&E) department in April, 2013 with history of a sudden onset of pain and swelling in the same knee. She heard a ‘pop’ in the knee while standing and did not report any obvious injury to the knee. On clinical examination, she was haemodynamically stable and afebrile and there was moderate effusion in the knee. The range of motion was from 30 to 60 degrees and she was unable to weight bear through the knee due to pain. The radiographs of her knee in the A&E department raised a suspicion of posterior dislocation of the polyethylene insert (Fig. 1). There was no evidence of loosening of femoral or tibial components.

**Fig. 2:**
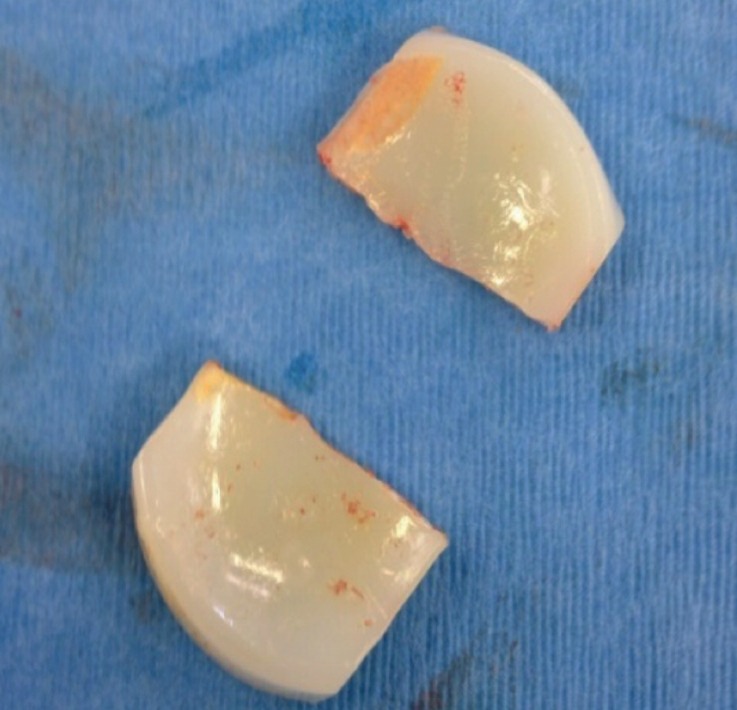
Retrieved bearing insert showing the clean fracture through the thinnest part and minimal wear of the polyethylene insert.

The white cell count and CRP were normal, excluding an acute infection. She was admitted to the ward and surgical exploration was planned for the following day, with the view of changing the polyethylene insert or to revise the components if they were loose or damaged. The knee was opened through the previous scar of medial para-patellar approach. Intraoperatively, both the femoral and the tibial components were found to be well fixed with no scratches and rest of the knee did not show any evidence of osteoarthritis. The polyethylene insert was found to be fractured through the middle (Fig. 2). The anterior half was sitting on the tibial component and the posterior half was dislodged into the posterior compartment of the knee, stuck to the posterior capsule. It was not possible to retrieve that fragment from the front.

**Fig. 3:**
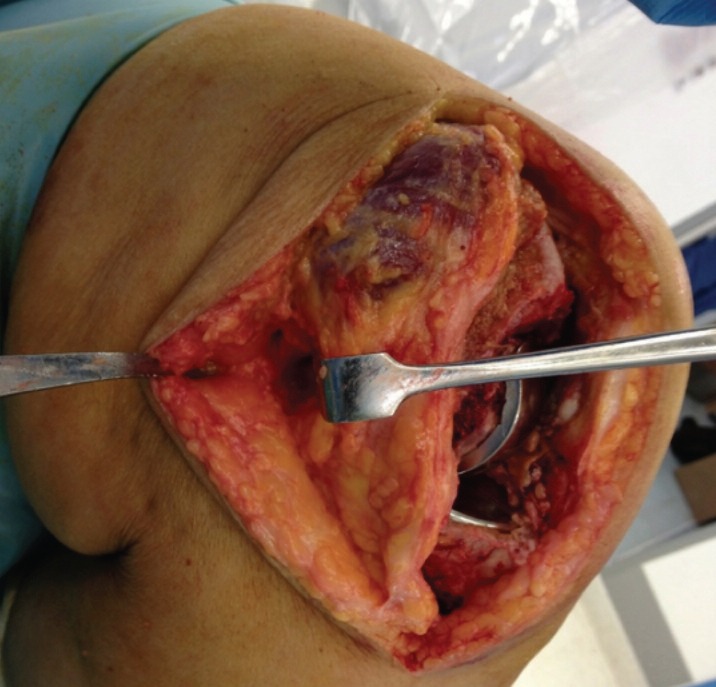
Intraoperative image showing raised medial skin flap and site of posteromedial capsulotomy.

The operating surgeon had two options; either to remove both the components and then retrieve the fragment from the front or to retrieve it through a posterior incision. Eventually, rather than making a separate skin incision, the previous skin incision was extended and medial skin flap was raised to expose the postero-medial aspect of the knee. Deep fascia was incised to expose the pes anserinus. An interval was created between the pes anserinus and the medial collateral ligament anteriorly and medial head of Gastronemius posterioly (Fig. 3). Capsulotomy was performed through this

interval and the dislodged part of the polyethylene insert was retrieved through the opening. The polyethylene insert showed some pitting and whitening and it seemed to have fractured cleanly through the thinnest part (Fig. 2). A new polyethylene of the same size insert was implanted. The patient made uneventful recovery following the surgery. At two year follow-up she was completely asymptomatic with the range of knee movement from 0 to 125 degrees.

## Discussion

Reported incidence of mobile bearing dislocation after medial unicompartmental knee arthroplasty ranges from 0 to 4%^1^. There are few reported cases of posterior dislocation of the meniscal bearing^4^ and only one reported case of fracture of the bearing in phase III Oxford UKA^3^.

Meniscal bearing dislocations in Oxford UKA usually occur due to failure in properly aligning the components or balancing the knee^1,4^. In some cases it could be because of impingement by osteophytes or due to intraoperative injury to the deep fibres of the medial collateral ligament, leading to laxity and bearing dislocation with trivial trauma^1,4^. Meniscal bearing in Oxford UKA is lipped anteriorly which makes its posterior dislocation extremely unlikely. If it does happen, posterior dislocation of the bearing insert could also be explained as being due to excessive posterior slope or damage to posterior structures while performing tibial resection, thereby making the knee lax posteriorly^4^.

If the knee is working normally with no impingement, the mean wear rate of the polyethylene insert is 0.01-0.02 mm/year^5^. Impingement due to loose bone or cement fragment results in high wear rate which has been shown to be the primary cause of fracture in all previous studies^3,5^. Oxidation (evident as whitening of the insert) and associated delamination has also been thought to be associated with fracture of the inserts^5^. The combination of high wear and oxidation results in thinning, initiation and propagation of cracks, resulting in fatigue fracture^5^.

In the present case, some pitting and whitening of the retrieved insert was obvious but there was no indication of excessive wear, contrary to previously reported cases^3,5^. The fracture line also looked very sharp and together with lack of excessive wear this indicated that the fracture in this case occurred due to sudden excessive load on a thin insert (3mm) which was weakened by oxidation. Diagnosis of the dislocation can be made by detailed history and clinical examination. AP and lateral radiographs of the knee joint are usually diagnostic but the fracture of the meniscal bearing can be missed as in this case.

It is important to recognise that rarely the fracture of the mobile bearing insert can occur even in the absence of excessive wear particularly when it is very thin. Many clinicians prefer to make the bone cuts thick enough to accommodate 4mm or thicker insert, hoping that it would avoid rare complication particularly in obese patients. Authors also suggest that the operating surgeon must be prepared for all the possibilities when planning the revision surgery in such cases: from just exchanging the mobile bearing insert to a revision total knee arthroplasty.

When faced with difficulty while retrieving the posteriorly dislocated meniscal bearing insert, one can perform a separate posteromedial capsulotomy through the same skin incision (as described above), which could avoid making a separate posterior skin incision (which may need repositioning of the patient) or avoid a more extensive surgery with revision of the components.

## Conflict of interest

The authors declare no conflicts of interest.
